# School-based one health education shapes young minds and households to prevent zoonoses: A comparison of four teaching strategies

**DOI:** 10.1016/j.onehlt.2026.101541

**Published:** 2026-08-13

**Authors:** Lorena Diniz Macedo Silva Maia, Breno Oliveira Lima Ramos, Marcelo Teixeira Paiva, Érica Lorenza Martins Araújo, Mariana Gomes Leal, Daniel Luiz de Miranda Cravo, Ana Clara Novaes, Isadora Martins Pinto Coelho, Clarice Lara Moreira, Ana Luisa Martins Brum, Rafael Romero Nicolino, Danielle Ferreira de Magalhães Soares, Camila Stefanie Fonseca de Oliveira

**Affiliations:** Department of Preventive Veterinary Medicine, School of Veterinary Medicine, Universidade Federal de Minas Gerais, Belo Horizonte, Minas Gerais, Brazil

**Keywords:** Elementary school students, One health, Health education, Zoonosis

## Abstract

Health education initiatives have received increasing attention in public policy; however, empirical evidence on the comparative efficacy of different teaching strategies remains limited. This study evaluated four educational approaches: group discussion, theater, board games, and traditional board-based instruction, among 166 elementary school students participating in activities focused on zoonoses, including rabies, sporotrichosis, Rocky Mountain spotted fever, and leishmaniasis. Adjusted exploratory analyses showed higher knowledge gain for board game than for group discussion, while no statistically significant differences were detected among the other strategies. Post-intervention scores were significantly higher than pre-intervention scores, indicating knowledge gains after the educational activities. The proportion of correct responses related to zoonoses increased from 15.7% to 56.6% (*p* < 0.05), while knowledge of One Health concepts rose from 38.2% to 55.7% (p < 0.05). Overall, the findings indicate that the educational interventions showed efficacy in enhancing students' knowledge and fostering behavioral changes within their family environments. These results highlight the potential of structured school-based health education to promote awareness and prevention of zoonotic diseases.

## Highlights

• Four teaching strategies were compared using a Latin square educational design.
• All strategies produced comparable improvements in students' zoonosis knowledge.
• Knowledge retention was assessed immediately, after one month, and at follow-up.
• Educational strategies may be adapted to students' age and school context.
• Schoolchildren can act as knowledge multipliers within their households.

## 1 Introduction

The health–disease process is shaped by interactions among humans, animals, and shared environments, including food production, work, domestic companionship, and ecosystem change [1], [2], [3]. Although the term “One Health” became widely used only in the twenty-first century, its conceptual roots lie in nineteenth-century comparative medicine, the study of zoonoses, and the recognition that human and animal diseases should not be understood within isolated disciplinary boundaries [2], [3]. This perspective was later consolidated by Schwabe's “One Medicine” paradigm, which proposed that there is no fundamental paradigmatic distinction between human and veterinary medicine, as both share biological, pathological, epidemiological, and population-health foundations [1], [2], [3]. From the late twentieth century onwards, this approach expanded to incorporate ecological, environmental, and social determinants of health, contributing to the “One World, One Health” framework and, more recently, to the current definition of One Health as an integrated and unifying approach that seeks to sustainably balance and optimize the health of people, animals, plants, and ecosystems [1], [2], [3].

Because One Health depends on coordinated action across sectors and communities, education is a key strategy for promoting prevention, awareness, and behavioral change. School-based interventions are particularly relevant because children have high learning potential and may disseminate knowledge within their families and communities [3]. From a One Health perspective, these interventions should address not only zoonotic diseases, but also animal behavior within ecosystems, responsible pet ownership, animal welfare, biodiversity conservation, and environmental health [4], [5]. This requires an ecocentric approach that moves beyond a human-centered framework and encourages students to recognize interdependence among human, animal, and ecosystem health. Such initiatives should therefore aim not only to transmit information, but also to foster critical thinking, autonomy, environmental responsibility, and problem-solving skills, empowering children to act as agents of change in their households and communities [4], [5].

Herd or population immunity shows how widespread individual protection can generate collective benefits by reducing disease transmission at the population level [6], [7]. Although educational interventions do not produce biological immunity, they may follow a similar logic: when preventive knowledge and health-promoting behaviors spread beyond directly exposed individuals, they can contribute to collective protection within families and communities [3], [6], [7]. Thus, children may influence the knowledge, attitudes, and behaviors of household members who did not participate directly in the activities, allowing school-based programs to produce broader and potentially lasting community impacts [3], [8].

Although health education with children is widely implemented, important gaps remain in the evaluation and comparison of teaching strategies used in these programs [9], [10], [11]. This gap is especially relevant in Veterinary Medicine and One Health education, where few studies have systematically assessed interventions addressing zoonoses, animal welfare, animal behavior within ecosystems, responsible pet ownership, and community-level health promotion.

Therefore, the primary objective of this study was to compare the efficacy of four educational strategies, delivered within a school-based health education project, in promoting knowledge about zoonoses and One Health among elementary school students. The secondary objectives were to evaluate students' and teachers' preferences for the strategies, investigate children's role as knowledge multipliers within their households, assess changes in family knowledge and practices, and identify barriers perceived by teachers when implementing alternative educational approaches.

## 2 Materials and methods

This community-based, non-randomized epidemiological study was conducted in 2024 in Contagem, Minas Gerais, Brazil, and was approved by the Research Ethics Committee of the Federal University of Minas Gerais (UFMG) (CAAE: 76846623.7.0000.5149).

### 2.1 Schools and participants

Schools were selected by the Municipal Department of Education through convenience sampling, considering feasibility and representation of different administrative regions [12]. All 3rd- and 7th-grade students in the selected schools were invited to participate, without individual-level sampling.

Participation required guardian consent and student assent. Students without signed parental or guardian consent would have been excluded from data collection but allowed to attend the educational activities. However, all invited students returned the required consent and assent forms and were included in the study. Thus, 166 students were invited and 166 participated, corresponding to a response rate of 100% among invited students. Guardians were eligible if they were adults, lived with or regularly interacted with the child, and provided informed consent. The selected grades were aligned with the Brazilian National Common Curricular Base, which introduces animal and environmental topics in the 3rd grade and expands to zoonoses, public health, and One Health in the 7th grade [13]. Students were aged 8–10 years in the 3rd grade and 11–14 years in the 7th grade, reflecting expected grade ages in Brazil and common age–grade variation in public schools [13].

### 2.2 Sample determination

The sample was determined by convenience and institutional feasibility, based on the number of eligible students enrolled in the selected 3rd- and 7th-grade classes in the participating schools. All eligible students in these classes were invited to participate. No a priori power calculation was conducted for between-strategy comparisons. Therefore, these comparisons were interpreted as exploratory, and the study was not powered to establish equivalence among strategies.

### 2.3 Topics addressed and strategies applied

This study addressed four zoonoses (rabies, sporotrichosis, Rocky Mountain spotted fever (RMSF), and visceral leishmaniasis) selected based on their prevalence or animal reservoirs in Contagem over the past five years [14]. For both 3rd and 7th-grade classes, activities covered definition, transmission, risk factors, clinical signs, diagnosis, prevention, and treatment.

A Latin square design was used to allocate the four educational strategies across classes, topics, and schools (Supplementary material 1) [15]. The allocation process was defined before the beginning of the interventions. First, the zoonosis topics to be addressed in each project session were randomly drawn, defining the topic for the first, second, third, and fourth sessions. Then, for the first session, the school that would receive each teaching strategy was randomly drawn. Based on this initial random allocation, the remaining allocations were determined systematically according to the Latin square structure, ensuring that all schools received all teaching strategies throughout the project and that strategies were balanced across sessions and topics. Each class participated in four thematic sessions and experienced all strategies, with each zoonosis taught through a different approach. This balanced design helped distribute the teaching strategies evenly across classes and topics, reducing the potential for confounding related to educational content, topic sequence, and school-specific characteristics. Although the selection of schools was not probabilistic, the allocation of topics and strategies within the Latin square design was defined before implementation and was not based on teacher, school, or researcher preference [15].

**Fig. 1:**
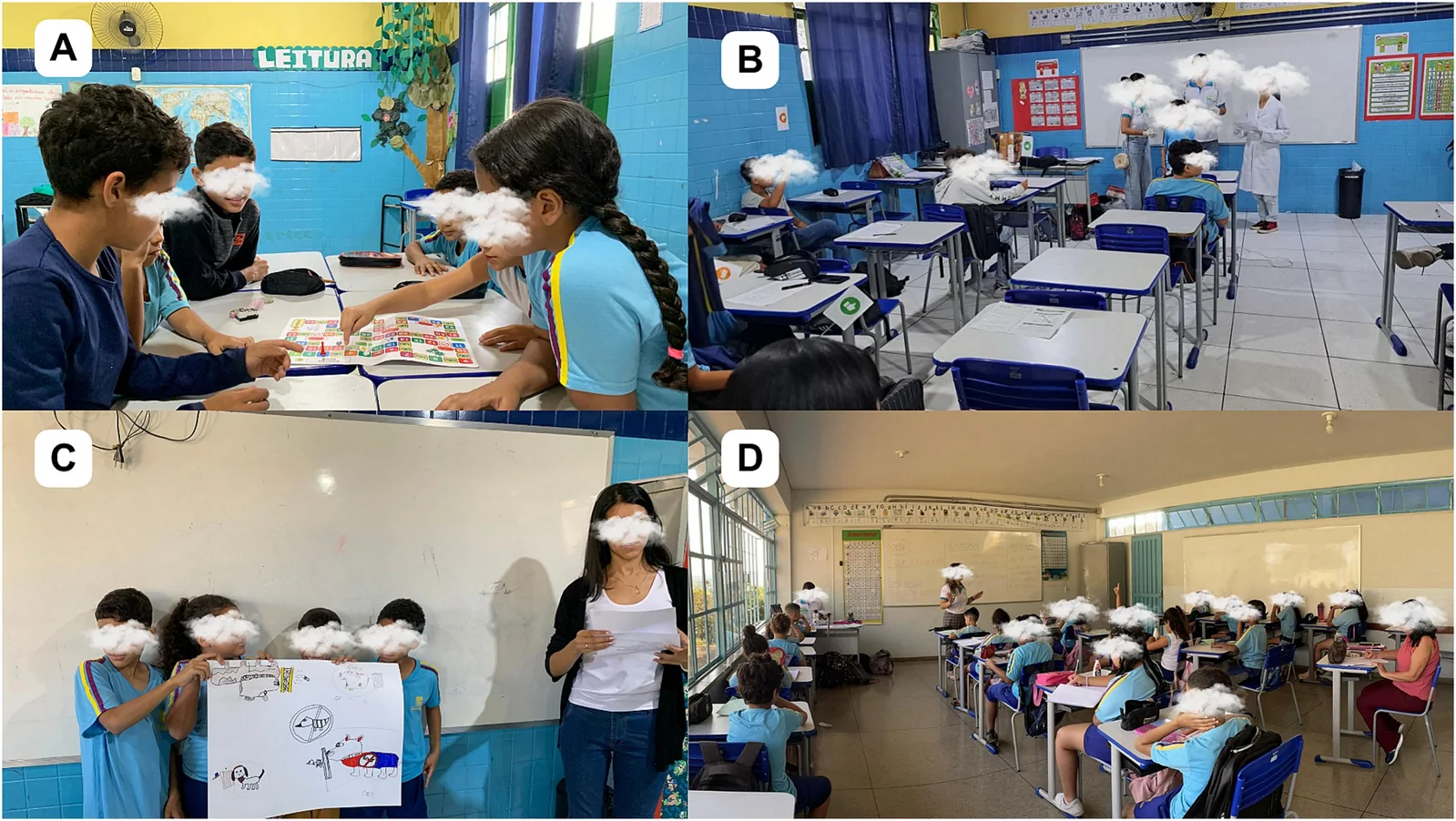
Teaching strategies used in the study, respectively A) board game, B) theater, C) group discussion, and D) board.

The board game used question-and-answer cards and a game board with reward and penalty spaces to teach zoonosis-related concepts interactively (Supplementary material 2, Supplementary material 3). Students played in groups, competing to reach the end while answering disease-related questions (Fig. 1A).

The theater strategy used 15-min scenes depicting everyday situations involving zoonotic disease risks, prevention, and the role of individuals in One Health (Fig. 1B). Performances involved the research team and one student from each class to increase engagement. The group discussion strategy promoted collaborative learning through a zoonosis-related problem. Students worked in groups of up to six to discuss the case, create posters illustrating prevention or control actions, and present their conclusions to the class (Fig. 1C). Posters were later displayed at school to disseminate preventive information. (See Table 1.)

**Table 1:** Sociodemographic profile of the students participating in the project by school, school year and gender.

Schools	Students n (%)	School year		Gender	
		3° N (%)	7° N (%)	Male n (%)	Female n (%)
Machado de Assis Municipal School (MA)	51 (30,7%)	22 (43,1%)	29 (56,9%)	28 (54,9%)	23 (45,1%)
Padre Joaquim de Souza Silva Municipal School (PJSS)	34 (20,5%)	19 (55,9%)	15 (44,1%)	20 (58,8%)	14 (41,2%)
Professor Hilton Rocha Municipal School (PHR)	49 (29,5%)	24 (49%)	25 (51%)	23 (46,9%)	26 (53,1%)
Professor Ricardo Braz Gomes Barreto Municipal School (PRBGB)	32 (19,3%)	15 (46,9%)	17 (53,1%)	16 (50%)	16 (50%)
Total	166 (100%)	80 (48,2%)	86 (51,8%)	87 (52,4%)	79 (47,6%)

Traditional board-based instruction was used as the control condition, representing a conventional teaching method rather than no intervention. Content was written on the board, copied by students, and followed by teacher-led explanation and questions (Fig. 1D). Before each session, a short introductory video presented the main zoonosis-related concepts using accessible language and visual elements (Supplementary Material 4).

Material costs ranged from BRL 12.50–46.00 (USD 2.50–9.20). Board game kits cost BRL 82.50 per class (USD 16.50), theater props BRL 21.00–40.00 (USD 4.20–8.00), and group discussion BRL 35.00 per class (BRL 7.00 per group; ≈USD 7.00). Video-based activities required no additional costs due to use of existing school equipment.

### 2.4 Student questionnaires

Students' learning was assessed using structured questionnaires administered immediately before and after each educational session. The instrument, developed for this study, included multiple-choice knowledge questions and Likert-scale perception items on interest, difficulty, learning, and motivation, with emoji-based response options to facilitate comprehension among younger students (Supplementary material 5). All questionnaires had the same structure, differing only in disease-specific content and review questions.

Knowledge questions addressed zoonoses, One Health, responsible pet ownership, animal welfare, and the disease covered in each session. Scores were calculated as the total number of correct answers, and learning gain was defined as the difference between post- and pre-activity scores. Retention was assessed using four disease-specific questions one month later, followed by a cumulative 44-question assessment five months after the first intervention, allowing evaluation of immediate, medium-term, and cumulative knowledge retention [3]. Baseline knowledge was reassessed before each session, and repeated exposure to previous topics was considered part of the educational process. Responses were collected using Google Forms, exported to spreadsheets, and analyzed as paired observations.

### 2.5 Questionnaires for teachers and guardians

After each session, teachers completed a questionnaire on the suitability, challenges, and limitations of the teaching strategies, covering teacher profile, teaching experience, motivation, use of alternative strategies, and class perception. Guardians completed questionnaires before and after the project to assess whether students disseminated the information learned at school. The questionnaires covered guardian profile, student engagement, animal health, student participation in the project, and project perception. Repeated sections allowed comparison of correct responses before and after the intervention.

### 2.6 Homework as a strategy to stimulate knowledge multiplication

After each class, students received topic-related homework to reinforce learning and encourage knowledge sharing with guardians, who were invited to complete it together. The activities were not graded and consisted of three sections: “Being a veterinarian for a day in my home!”, in which students and guardians used a checklist to identify potential disease risks in the household; “Shoo, (disease)! Look what we can draw”, a space for drawing a disease prevention measure; and “Fighting (disease)”, a word search or “spot the differences” activity related to the topic. Completed homework was collected by teachers and delivered to the project team during the following session.

### 2.7 Interventions

Each intervention was conducted by two to five trained members of the research team and followed a standardized sequence: greeting and explanation of the activity, pre-activity questionnaire completion, presentation of a short introductory video on the zoonosis addressed, implementation of the main educational activity, post-activity questionnaire completion, and distribution of topic-related homework. Questionnaires took approximately eight–10 min before the activity and five minutes afterward, while the main activity lasted 25–30 min.

### 2.8 Focus group

At the end of the project, focus groups were conducted to qualitatively explore students' experiences, perceptions of the educational strategies, knowledge sharing with guardians, and perceived changes at home. These sessions complemented the quantitative findings and were not designed to formally assess socio-emotional competencies.

Ten focus groups were conducted, corresponding to one per class, with larger classes divided when necessary to maintain the recommended group size of 8–15 students. Sessions lasted 35–50 min, were conducted in the classroom without teachers present, and included all participating students who were present on the day of data collection. Each session was facilitated by a moderator and an assistant using a semi-structured script and established focus group procedures [16].

Participants were informed about the objectives, voluntary participation, confidentiality, and audio recording. Sessions began with a brief recap of the diseases and educational strategies, followed by guided discussion. Recordings were transcribed verbatim and analyzed to identify central themes and students' key perceptions.

### 2.9 Data preparation and statistical analyses

Data were cleaned and organized using unique student codes. Incomplete student questionnaires and guardian responses from individuals under 18 years of age were excluded, resulting in a final sample of 166 students, eight teachers, and 91 guardians.

Knowledge gain was calculated as the difference between post- and pre-intervention scores. The primary analysis of knowledge gain was performed using a linear mixed-effects model [17]. Teaching strategy, session, and school were included as fixed effects, and student was included as a random intercept to account for repeated measurements within students. An initial model including both student and school as random intercepts was fitted to evaluate school-level clustering. In this model, the school-level variance was estimated as zero, which suggests that, after accounting for teaching strategy, session, and repeated measurements within students, the model detected no additional variability in knowledge gain attributable to differences between schools. However, because only four schools participated, the school-level variance and ICC could not be estimated precisely. For this reason, school was included as a fixed effect in the final model.

Estimated marginal means were used to compare adjusted knowledge gains among teaching strategies and sessions, with Tukey adjustment for pairwise comparisons. Additional mixed-effects models were used to evaluate pre- and post-intervention scores by grade and teaching strategy. Model assumptions were assessed using residual-versus-fitted plots and quantile–quantile plots, supplemented by formal tests of residual normality and homoscedasticity. The residuals showed no substantial departure from normality, and their variance was approximately constant across fitted values, with the formal tests providing no evidence of violations of these assumptions.

Descriptive analyses were used to summarize student characteristics, pet ownership, frequencies of correct answers for specific knowledge items, and overall pre- and post-intervention performance. For categorical outcomes and questionnaire responses from teachers and guardians, proportions were compared using pairwise proportion tests, and associations between categorical variables were assessed using Pearson's chi-square or Fisher's exact tests, as appropriate. Analyses were conducted in RStudio [18], adopting a 5% significance level.

## 3 Results

A total of 166 students participated, including 86 from the 7th grade (51.8%) and 80 from the 3rd grade (48.2%), aged 8–14 years; 52.4% were female. Most students owned pets (76.2%), mainly dogs (60.9%) and cats (18.0%).

**Fig. 2:**
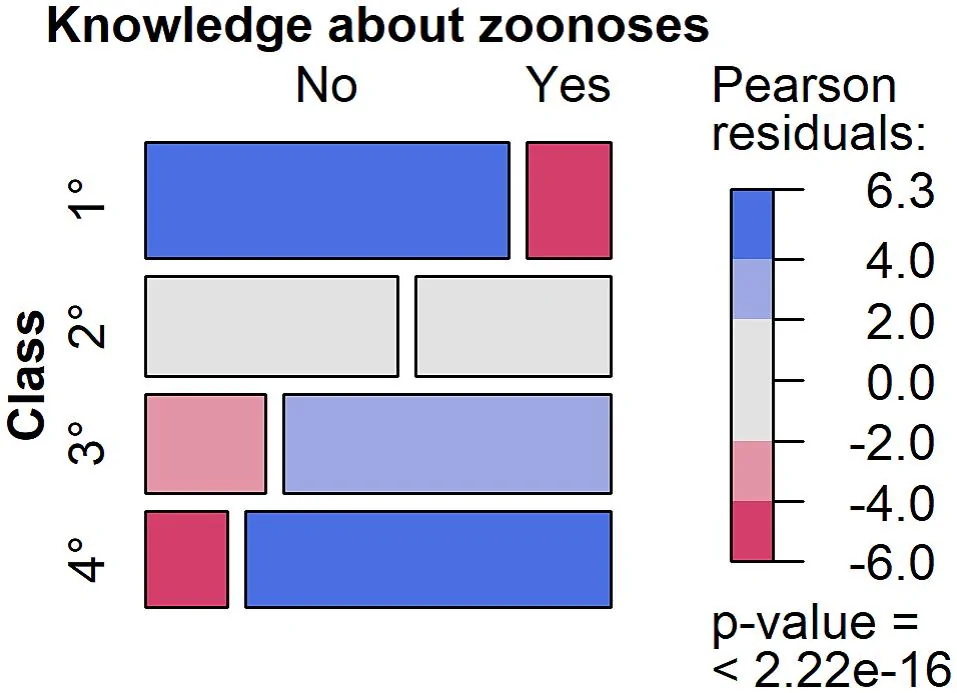
Mosaic plot of the standardized Pearson residuals* with the *p*-value of the association between “knowledge about zoonoses” and “assessment moment” during the school-based health education project.

Correct answers on zoonoses increased from 15.7% to 56.6%, representing a 40.9 percentage-points increase and a 3.6-fold relative improvement (*p* < 0.01) (Fig. 2).

**Fig. 3:**
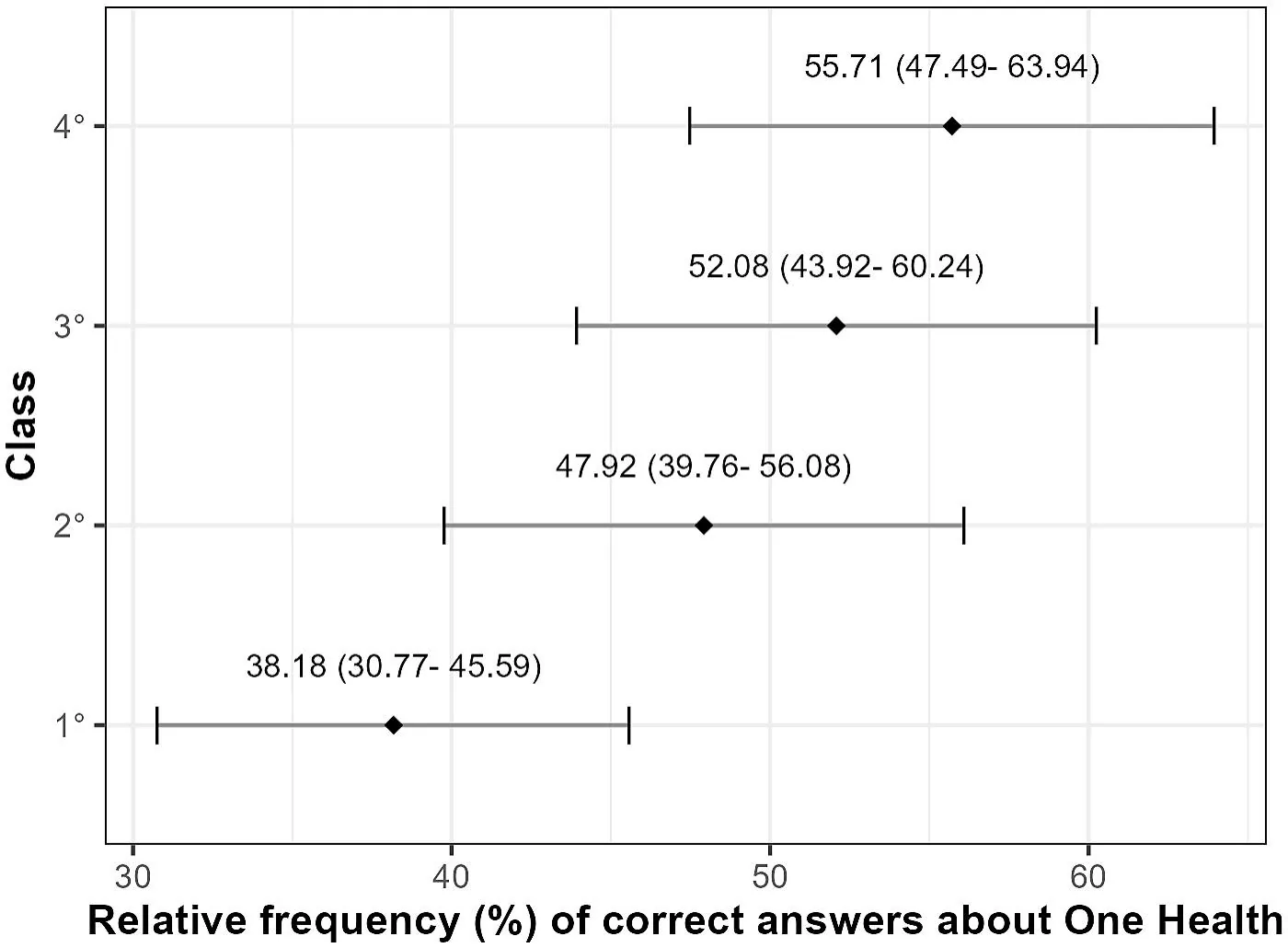
Relative frequency (%) with a 95% confidence interval regarding correctly identifying the “One Health” concept by class during the project.

One Health knowledge also increased, from 38.2% to 55.7%, representing a 17.5 percentage-point increase and a 1.46-fold relative improvement (p < 0.01) (Fig. 3).

Knowledge of responsible pet ownership increased from 72.2% to 85.3%, although this change was not significant (*p* > 0.05). Students without pets scored higher (88.06%) than those with pets (79.19%) (*p* < 0.05).

**Fig. 4:**
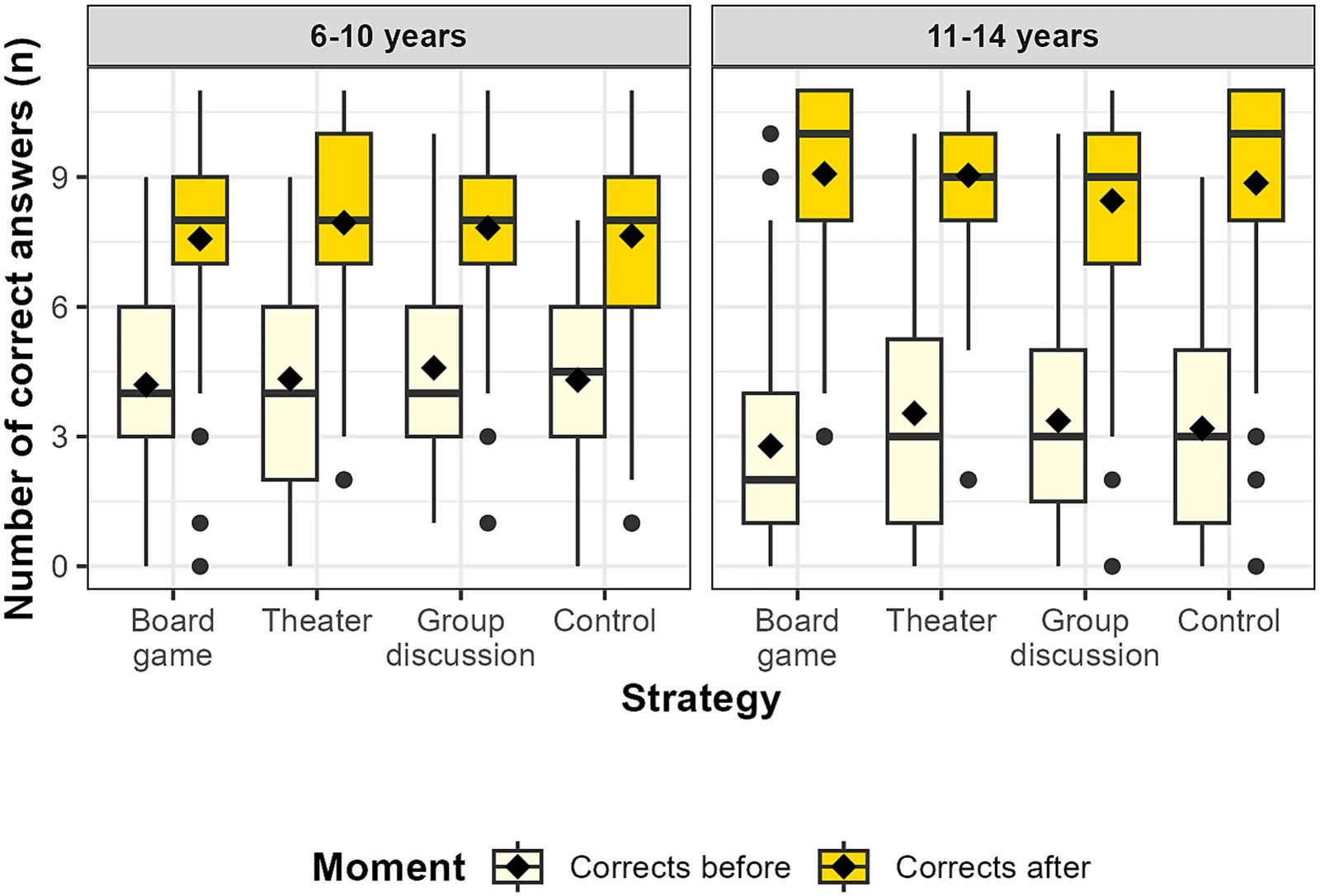
Boxplot of the number of correct answers before and immediately after the interventions among 3rd-grade students aged 8–10 years and 7th-grade students aged 11–14 years, respectively.

Post-intervention scores were significantly higher than pre-intervention scores in both grades (p < 0.01) (Fig. 4).

The mixed-effects analysis of knowledge gain included 595 student-session observations with complete pre- and post-intervention data: 166 in session 1, 144 in session 2, 145 in session 3, and 140 in session 4. In the initial random-intercept model including both student and school, the school-level variance was estimated as zero, resulting in a school-level ICC of 0.000. Because only four schools participated, this estimate was interpreted cautiously, and school was included as a fixed effect in the final model. In the adjusted model, estimated marginal mean knowledge gain was highest for the board game strategy (4.83; 95% CI: 4.33–5.33), followed by theater (4.58; 95% CI: 4.08–5.08), traditional board-based instruction (4.54; 95% CI: 4.04–5.04), and group discussion (4.05; 95% CI: 3.53–4.57). Pairwise comparisons showed a higher adjusted gain for board game than for group discussion (estimated difference = 0.78 points; Tukey-adjusted *p* = 0.033). No statistically significant differences were detected among the other strategies.

Adjusted knowledge gain also varied across sessions. Gains were higher in session 1 than in sessions 2 and 4, and higher in session 3 than in session 4. In the model evaluating scores before and after the interventions, adjusted mean scores increased significantly in both grades, from 4.34 to 7.73 in the 3rd grade (*p* < 0.05) and from 3.17 to 8.83 in the 7th grade (p < 0.05). The estimated gains were 3.39 and 5.66 points, respectively, indicating greater knowledge gain among 7th-grade students. The interaction between assessment moment and teaching strategy was not statistically significant, indicating that no clear difference in pre–post gain among teaching strategies was detected in this exploratory analysis.

Students reported similar satisfaction across strategies, with no significant differences in preference, motivation, perceived difficulty, or content assimilation (*p* > 0.05) (Table 2). Group-based activities were preferred by both younger (89.9%) and older (87.6%) students. Focus groups supported these findings, as students described interactive strategies as engaging and participatory, while traditional board-based instruction was associated with copying and lower interaction. Students also reported sharing zoonosis prevention information with guardians and encouraging preventive practices at home, such as yard cleaning, pet vaccination, repellent collar use, and restricting animal roaming (Supplementary material 6).

Eight teachers participated, most of whom were female (75%) and aged 35–50 years. Group discussion was the preferred strategy, receiving the highest rankings for stimulating student interest (32%), promoting learning (29.2%), cost–benefit ratio (32%), and alignment with educational innovations (32%). Theater and board games were also frequently highlighted, particularly for increasing student interest (24%) and supporting learning (25%). For teacher learning, theater, board games, and group discussion received similar rankings. In contrast, traditional board-based instruction was the least preferred strategy across most categories.

**Table 2:** Strategies classification in students perspective in categories “Cool/very cool”, “Easy/very easy”, “Learned a lot/too much” and “Motivated/very motivated”, according to age, disposted in relatives frequency (%) (95% CI) and significance of ratio testing in pairs.

Classification	Teaching strategy	Age			
		Six to 10 years		11 to 14 years old	
		Relative frequency (%) 95% CI	Ratio Testing in Pairs	Relative frequency (%) 95% CI	Ratio Testing in Pairs
Cool/very cool	Board Game	67/69 (97.1%, CI = 93.14–100)	a*	79/81 (97.5%, CI = 94.15–100)	a*
	Theater	71/72 (98.6%, CI = 95.91–100)	a	80/80 (100%, CI = 100–100)	a
	Group discussion	68/68 (100%, CI = 100–100)	a	70/70 (100%, CI = 100–100)	a
	Board	69/72 (95.8%, CI = 91.22–100)	a	79/80 (98.8%, CI = 96.32–100)	a
Easy/very easy	Board Game	61/69 (88.4%, CI = 80.85–95.96)	a*	79/82 (96.3%, CI = 92.28–100)	a*
	Theater	67/72 (93.1%, CI = 87.18–98.93)	a	77/80 (96.2%, CI = 92.09–100)	a
	Group discussion	67/68 (98.5%, CI = 95.67–100)	a	68/70 (97.1%, CI = 93.24–100)	a
	Board	64/71 (90.1%, CI = 83.21–97.08)	a	74/79 (93.7%, CI = 88.3–99.04)	a
Learned a lot/too much	Board Game	68/69 (98.6%, CI = 95.73–100)	a*	77/82 (93.9%, CI = 88.72–99.08)	a*
	Theater	70/72 (97.2%, CI = 93.43–100)	a	80/80 (100%, CI = 100–100)	a
	Group discussion	65/68 (95.6%, CI = 90.71–100)	a	67/68 (98.5%, CI = 95.67–100)	a
	Board	70/72 (97.2%, CI = 93.43–100)	a	75/80 (93.8%, CI = 88.45–99.05)	a
Motivated/ very motivated	Board Game	64/69 (92.8%, CI = 86.64–98.87)	a*	76/82 (92.7%, CI = 87.05–98.32)	a*
	Theater	67/72 (93.1%, CI = 87.18–98.93)	a	71/80 (88.8%, CI = 81.83–95.67)	a
	Group discussion	63/68 (92.6%, CI = 86.44–98.85)	a	66/70 (94.3%, CI = 88.85–99.72)	a
	Board	65/72 (90.3%, CI = 83.43–97.12)	a	69/79 (87.3%, CI = 80.01–94.67)	a

* Equal letters indicate statistically equal groups on the Pair Proportion Test with 5% significance

Ninety-one guardians participated, representing 54.8% of student guardians; most were female (82.4%), mothers (74.7%), and guardians of 3rd-grade students (56%). Board games generated the greatest reported interest, particularly for rabies and leishmaniasis. In total, 404 homework assignments were completed, mostly by younger students (60%), who more often requested guardian support and applied the content at home.

By the end of the project, guardians perceived students as knowledge multipliers: 83.3% reported effective information sharing, 76.2% stated they had learned from the children, and 52.4% reported behavioral changes, mainly related to responsible pet care and preventive measures. Guardian knowledge increased significantly for RMSF and rabies (*p* < 0.05), but not for sporotrichosis or leishmaniasis (*p* > 0.05). The greatest gain was observed for RMSF, with correct responses increasing from 16.48% (95% CI: 8.86–24.11) to 87.5% (95% CI: 77.25–97.75). Rabies knowledge also increased, from 71.43% (95% CI: 62.15–80.71) to 92.86% (95% CI: 85.07–100).

## 4 Discussion

Students' knowledge increased significantly after interventions. In the adjusted exploratory analysis, the board game showed higher knowledge gain than group discussion, while no statistically significant differences were detected among the other strategies. However, this difference was modest, corresponding to less than one additional point in adjusted knowledge gain. Therefore, the finding should be interpreted cautiously and should not be used alone to recommend one strategy over another. In school-based health education projects, strategies should be selected according to learning objectives, feasibility, cost, accessibility, educational context, and target audience. Further studies are needed to determine whether any strategy is equivalent or superior to another.

Most students owned pets, reflecting the high prevalence of companion animals in Brazilian households, where approximately 70% of households have animals [19]. However, students without pets performed better on responsible pet ownership questions, suggesting that living with animals does not necessarily imply accurate knowledge or responsible care practices. Children may reproduce household practices misaligned with animal welfare, such as free roaming or neglect of preventive care. Thus, pet ownership should not be considered a proxy for responsible guardianship knowledge. This is consistent with the vulnerability of companion animals in Brazil, where approximately 25% of dogs and cats are estimated to live without a home, reinforcing the need for school-based programs addressing animal welfare and responsible pet ownership, especially among children who already interact with animals at home [20], [21].

Performance varied by age group, which should guide pedagogical planning. According to Piaget's theory, younger students are generally in the concrete operational stage and benefit from concrete examples, whereas older students can engage more with abstract reasoning [22], [23], [24]. Therefore, zoonosis prevention, One Health, and responsible pet ownership may require grade-specific conceptual adaptation. Younger students also showed greater engagement in homework, often requesting guardian support and sharing classroom content at home. This aligns with Vygotsky's Zone of Proximal Development, in which learning is strengthened through guided interaction with more experienced individuals [25]. Joint homework may therefore reinforce learning while promoting family engagement [23], [25].

Guardians' knowledge varied across diseases. Knowledge about leishmaniasis remained limited despite the disease being established in the municipality [26], [27], while sporotrichosis knowledge did not significantly improve, possibly due to previous local awareness campaigns since its emergence in the region [28]. Nevertheless, most guardians reported learning new information and implementing changes at home. In contrast, knowledge about RMSF increased markedly, from 16.48% to 87.5%, the greatest gain observed, suggesting that school-based health education may be especially valuable for neglected zoonoses with low baseline awareness. Given that Contagem reported 384 RMSF cases between 2019 and 2023 [14], this knowledge gap is concerning. Educational initiatives involving children and families may improve community awareness and preventive behaviors, particularly for zoonoses that remain underestimated despite local surveillance efforts [29], [30], [31], [32].

Students often preferred group-based activities, highlighting the value of collaborative learning in health education. Such approaches encourage dialogue, exchange of ideas, argumentation, and communication skills [33], [34]. Focus groups supported the quantitative results, as students perceived board games, theater, and group discussion as more engaging and participatory than traditional board-based instruction [16]. Students also reported sharing information with guardians and encouraging preventive practices at home, suggesting that school-based interventions may extend beyond individual learning and contribute to household-level awareness.

Teachers considered group discussion the most favorable strategy for stimulating interest, promoting learning, and aligning content with educational innovations. This preference was supported by feasibility, as group discussion requires minimal resources, has low implementation costs, approximately USD 7.00 per class, and can be adapted to different topics without specialized infrastructure. Thus, it may be suitable for resource-limited health education projects, although feasibility depends on classroom conditions, available time, planning, and student engagement. In contrast, traditional board-based instruction, although widely used, may limit interaction because much class time is devoted to copying content, resulting in passive knowledge transmission and reduced collaborative learning [35]. Although the strategies were delivered by the research team, teachers' perspectives were relevant because they are key stakeholders in the school environment and can help identify practical conditions, barriers, and adaptations needed for future health education projects conducted in partnership with schools.

Competition, a central element of the board game, may increase student engagement, and appropriately structured gamification can enhance motivation and participation [36]. Similarly, students value dialogue and opportunities to consider different viewpoints, while educator-mediated discussions can improve learning and engagement [13], [37], [38]. Therefore, interactive and student-centered strategies should be incorporated into health education, with teachers' perspectives considered in project design.

Despite the importance of One Health, animal welfare, responsible pet ownership, and zoonoses, these topics remain largely absent from the Brazilian national curriculum. A recent review identified no explicit references to “animal health” or “zoonoses” and only limited mentions of “public health” and “prevention”, highlighting the need to integrate health education earlier into school curricula [13]. These findings reinforce that schools should go beyond technical instruction and contribute to the development of social and ethical values, as proposed in the pedagogical framework [13], [37], [38].

The intervention incorporated internationally recognized One Health principles by addressing the interdependence between human, animal, and environmental health in the context of zoonotic diseases. All approaches contributed to awareness and preventive practices within the context of the health education project, although adjusted analyses detected a higher gain for board game compared with group discussion. Students shared knowledge with parents or guardians, discussed prevention at home, and sometimes encouraged behavioral changes. This transfer of knowledge beyond the classroom demonstrates community engagement, an important component of the One Health approach [4], [5]. The intervention also aligned with education for global citizenship and ecosystem health by promoting awareness that human health is connected to animal health and environmental integrity. Activities addressed responsible pet ownership, animal welfare, household environmental management, and shared responsibility for zoonosis prevention, fostering an ecocentric perspective and reinforcing One Health relevance for disease prevention, responsible citizenship, and sustainable health practices [4], [5].

Some limitations should be acknowledged. Although the Latin square design reduced topic-related confounding and included a traditional board-based control condition, the selection of schools was not probabilistic, and selection bias or residual confounding cannot be excluded. The adjusted analysis accounted for school by including it as a fixed effect, however, only four schools participated. In the initial random-intercept model, school-level variance was estimated as zero and the school-level ICC was 0.000. Therefore, the findings should still be interpreted cautiously, as the small number of schools limits broader inference about school-level effects. As all students received an intervention, the absence of a non-intervention control group limits causal inference, since repeated testing, external information, and cognitive maturation may have contributed to knowledge gains.

Successive sessions may also have introduced carryover, order effects, and cumulative learning. Because each class received all four teaching strategies in sequence, students' performance in later sessions may have been influenced by knowledge acquired in earlier sessions, greater familiarity with the project format, and repeated exposure to questionnaire procedures, regardless of the teaching strategy used. Adjusted analyses showed that knowledge gain varied across sessions, supporting the possibility that the intervention sequence influenced students' performance. Gains were lower in later sessions, particularly session 4, which may reflect reduced novelty, fatigue, or cumulative learning increasing baseline knowledge and reducing the margin for additional immediate gain. Conversely, repeated exposure to project content and questionnaire procedures may also have facilitated responses in later sessions. Therefore, comparisons among strategies should be interpreted as exploratory findings from real school conditions rather than definitive causal estimates. The absence of statistically significant differences in most between-strategy comparisons should be understood as no clear difference detected in this study, rather than as evidence that the strategies are equivalent.

Additional limitations include difficulties with some closed-ended questions among younger students, delivery of all strategies by trained members of the research team rather than regular classroom teachers, lack of socioeconomic and academic data, absence of follow-up beyond five months, limited guardian engagement, and use of non-validated questionnaires. Because all strategies were researcher-delivered, this study provides evidence of efficacy under standardized intervention conditions, rather than evidence of effectiveness when implemented by teachers in routine school practice. Future studies should evaluate teacher-delivered implementation, including teacher training, adaptation to school routines, and fidelity monitoring. Thus, focus group findings should be interpreted as reported experiences rather than validated measures of competency development.

## 5 Conclusion

The school-based health education project showed efficacy in improving students' knowledge of zoonoses and One Health. Adjusted exploratory analyses indicated higher knowledge gain for the board game than for group discussion, while no statistically significant differences were detected among the other strategies. However, this finding should be interpreted cautiously, and strategy selection should also consider educational context, available resources, feasibility, cost, accessibility, and target audience. Students also acted as knowledge multipliers, sharing information beyond the classroom and increasing awareness within their families. Guardians reported learning from their children, especially regarding RMSF, highlighting the potential of school-based interventions to address neglected zoonoses with low baseline awareness. Overall, school-based health education can operationalize internationally recognized One Health principles while promoting knowledge dissemination, preventive practices, and community engagement.

## CRediT authorship contribution statement

**Lorena Diniz Macedo Silva Maia:** Writing – review & editing, Writing – original draft, Visualization, Validation, Project administration, Methodology, Investigation, Formal analysis, Data curation, Conceptualization. **Breno Oliveira Lima Ramos:** Writing – original draft, Resources, Investigation. **Marcelo Teixeira Paiva:** Software, Formal analysis, Conceptualization. **Érica Lorenza Martins Araújo:** Resources, Methodology, Investigation, Conceptualization. **Mariana Gomes Leal:** Resources, Methodology, Investigation, Conceptualization. **Daniel Luiz de Miranda Cravo:** Resources, Methodology, Investigation. **Ana Clara Novaes:** Resources, Methodology, Investigation. **Isadora Martins Pinto Coelho:** Methodology, Investigation, Formal analysis. **Clarice Lara Moreira:** Resources, Methodology. **Ana Luisa Martins Brum:** Resources, Investigation, Conceptualization. **Rafael Romero Nicolino:** Writing – review & editing, Formal analysis, Conceptualization. **Danielle Ferreira de Magalhães Soares:** Writing – review & editing, Methodology, Data curation, Conceptualization. **Camila Stefanie Fonseca de Oliveira:** Writing – review & editing, Validation, Supervision, Resources, Project administration, Methodology, Funding acquisition, Formal analysis, Data curation, Conceptualization.

## Ethics approval and consent to participate

This study was approved by the Research Ethics Committee of the Federal University of Minas Gerais (UFMG), Brazil (CAAE: 76846623.7.0000.5149). Participation was voluntary and required written informed consent from parents or legal guardians and assent from students. Guardians who participated in the study also provided informed consent.

## Ethics declaration

### Informed consent and patient details

Written informed consent to take part in the study and to publish the article has been obtained from all participants or their legal representatives. The privacy rights of participants have been observed.

### Studies in human

This study was performed in compliance with relevant laws, regulatory frameworks and guidelines where the research took place.

This study was approved by the Research Ethics Committee of the Federal University of Minas Gerais.

(Approval No. 76846623.7.0000.5149)

## Funding

The authors acknowledge the support of CAPES (Coordenação de Aperfeiçoamento de Pessoal de Nível Superior, Brazil). CAPES had no role in the study design, data collection, analysis, interpretation of data, writing of the manuscript, or decision to submit the article for publication.

## Declaration of competing interest

The authors declare that they have no known competing financial interests or personal relationships that could have appeared to influence the work reported in this paper.

## Data Availability

The data that support the findings of this study are not publicly available due to ethical restrictions involving data collected from children and guardians. Anonymized data may be made available by the corresponding author upon reasonable request and subject to approval by the appropriate ethics committee.

## Appendix A Supplementary data

Supplementary material 8

Supplementary material 9 Proof of English Language Revision
