# Supplementary material for: School-based one health education shapes young minds and households to prevent zoonoses: A comparison of four teaching strategies
Source: One Health. 2026 Aug 13;23:101541. doi: 10.1016/j.onehlt.2026.101541 (PMC13519258; doi:10.1016/j.onehlt.2026.101541)
Supplement: Supplementary file 1 [file mmc9.pdf]

Proof of English Language Revision

This document certifies that the manuscript entitled "When does health education shape young minds and homes to prevent zoonoses?" has undergone thorough English language revision, including grammar, syntax, style, and overall clarity.

The revision was conducted by Isadora Martins Pinto Coelho, PhD student and Research Assistant at Iowa State University, and co-author of this study.

The authors confirm that the text has been carefully checked and corrected to improve readability, coherence, and linguistic accuracy.

Isadora Martins Pinto Coelho

Isadora Martins Pinto Coelho

March 23, 2026

Documento assinado digitalmente
gov.br
ISADORA MARTINS PINTO COELHO
Data: 23/03/2026 18:34:51-0300
Verifique em https://validar.iti.gov.br
