# Supplementary material for: School-based one health education shapes young minds and households to prevent zoonoses: A comparison of four teaching strategies
Source: One Health. 2026 Aug 13;23:101541. doi: 10.1016/j.onehlt.2026.101541 (PMC13519258; doi:10.1016/j.onehlt.2026.101541)
Supplement: Supplementary file 2 [file mmc4.docx]

**Supplementary materials 4**
**Videos**

Supplementary material 4. Access links to the educational videos used during the intervention, covering sporotrichosis (A), leishmaniasis (B), Rocky Mountain spotted fever (C), and rabies (D).

(A) Sporotrichosis. <https://youtu.be/bmy_tSbDryU?si=If_ZKtK-rV7Q_VYb>

<https://youtu.be/bmy_tSbDryU?si=If_ZKtK-rV7Q_VYb>

(B) Leishmaniasis: <https://www.youtube.com/watch?v=gMGdVvFJQns>

<https://www.youtube.com/watch?v=gMGdVvFJQns>

(C) Rocky Mountain spotted fever: <https://www.youtube.com/watch?si=JgFodrynBdkB29_r&v=kh0JoVaercA&feature=youtu.be>

<https://www.youtube.com/watch?si=JgFodrynBdkB29_r&v=kh0JoVaercA&feature=youtu.be>

(D) Rabies: <https://www.youtube.com/watch?v=VcZvIW9nYw0>

<https://www.youtube.com/watch?v=VcZvIW9nYw0>
