# Supplementary material for: School-based one health education shapes young minds and households to prevent zoonoses: A comparison of four teaching strategies
Source: One Health. 2026 Aug 13;23:101541. doi: 10.1016/j.onehlt.2026.101541 (PMC13519258; doi:10.1016/j.onehlt.2026.101541)
Supplement: Supplementary file 3 [file mmc5.docx]

**Supplementary Material 5**

**Student Questionnaire**

Supplementary Material 5. Student questionnaire administered during the final educational session. The questionnaire included items on the topic of the day (rabies), review questions from previous sessions, concepts of One Health, responsible pet ownership, animal welfare, and students’ perceptions of the educational activity.

Hello everyone! We are from UFMG and we are going to do a project at your school about animal diseases. You will answer these questions now and others after class. We will have a very fun class! **Let's go?**

**BEFORE CLASS**

1. **Full Name:** ____________________________________________________________
2. **What year are you from?** (_) 3rd year (_) 7th year
3. **School:**
   (_) Padre Joaquim de Souza Silva Municipal School (_) Professor Hilton Rocha Municipal School (_) Professor Ricardo Braz Gomes Barreto Municipal School (_) Machado de Assis Municipal School

**Knowing about animal health**

1. Do you know what **"zoonoses" are**?
   a. Yes b. No
2. **What are zoonoses?** Mark with an X the sentence that explains what it is (mark only 1 sentence).
   a. Diseases we get from spoiled food
   b. Diseases we get from dirty water
   c. Diseases that appear in terrestrial domestic animals
   d. Diseases that are passed between people and animals
   e. I don't know what zoonoses are
3. Do you think **that the health of animals has to do with the health of people and nature**? Mark with an X the sentence you think is right (mark only 1 sentence).
   a. No, because animals and people have different diseases
   b. No, because nature does not catch diseases and does not die either
   c. Yes, but only if the sick animal is inside our home
   d. Yes, if animals are sick, people and nature are at risk
   e. I can't say if there is a relationship between the health of animals, people and nature
4. The phrases below talk about animal welfare, **which phrases do you believe are right?** Mark with an X the phrases that you think are right (you can mark one more sentence)
   a. (_) Dogs need the kibble more than cats, as cats go out on the street to hunt for their own food
   b. (_) Dogs should be leashed all day and should only be released at night
   c. (_) Vaccinating dogs and cats is important to prevent them from having diseases, such as rabies, for example
   d. (_) It is important that dogs and cats have a shelter to be able to hide from the rain and the strong sun
   e. (_) Dogs and cats do not stress or feel fear, because they are animals and animals do not have feelings
5. Do you think **cats and dogs can leave the house alone**? Mark with an X the sentence you think is right (mark only 1 sentence).
   a. Yes, because they are free animals and need to go out hunting
   b. Yes, because they need to walk on the street alone so they don't get stressed
   c. No, because they need to be locked indoors or in the backyard
   d. No, because they can get diseases, fight or be run over

**PREVIOUS CLASSES**

**Remembering the last class: "Spotted Fever"**

Read the following statements, and in each of them, mark whether you consider it to be true or false. If you don't know the answer, check the "I don't know" option. It's okay not to know or make mistakes!

1. "People and dogs can have spotted fever".
   a. Truth	b. Lie	c. I don't know
2. "I must wear a black T-shirt whenever I go to a park or a place that has grass or bush to be protected from spotted fever."
   a. Truth	b. Lie	c. I don't know
3. "There is no treatment or medicine for spotted fever, so all people with the disease die."
   a. Truth	b. Lie	c. I don't know
4. "The only two ways to transmit spotted fever are through the bite of the capybara and the bite of the star tick."
   a. Truth	b. Lie	c. I don't know

**Remembering the second class: "Leishmaniasis"**

Read the following statements, and in each of them, mark whether you consider it to be true or false. If you don't know the answer, check the "I don't know" option. It's okay not to know or make mistakes!

1. "People and dogs can have leishmaniasis."
   a. Truth	b. Lie	c. I don't know
2. "Dogs that walk at night are more at risk of catching the disease than dogs that walk in the morning."
   a. Truth	b. Lie	c. I don't know
3. "There is no treatment for leishmaniasis, so all dogs with the disease must die."
   a. Truth	b. Lie	c. I don't know
4. "The most common way to have leishmaniasis is through the bite of the dengue mosquito (*Aedes aegypti*)".
   a. Truth	b. Lie	c. I don't know

**Remembering the first class: "Sporotrichosis"**

Read the following statements, and in each of them, mark whether you consider it to be true or false. If you don't know the answer, check the "I don't know" option. It's okay not to know or make mistakes!

1. "People and cats can get sporotrichosis"
   a. Truth	b. Lie	c. I don't know
2. "If my kitten has sporotrichosis, he should go for a walk on the street to be less stressed and get better faster."
   a. Truth	b. Lie	c. I don't know
3. "All cats that have the disease sporotrichosis should be euthanized (die), since this disease has no cure."
   a. Truth	b. Lie	c. I don't know
4. "The most common way to have sporotrichosis is through the mosquito bite"
   a. Truth	b. Lie	c. I don't know

**TODAY'S CLASS**

**Knowing about "Rabies"**

Read the following statements, and in each of them, mark whether you consider it to be true or false. If you don't know the answer, check the "I don't know" option. It's okay not to know or make mistakes!

1. Have you ever heard about anger?
   a. Yes	b. No	c. I don't know
2. "People, dogs and cats can get rabies".
   a. Truth	b. Lie	c. I don't know
3. "There is no treatment or remedies as efficient for rabies, so almost 100% of people with the disease die."
   a. Truth	b. Lie	c. I don't know
4. "The only symptom that people or animals with the disease 'rabies' have is aggressiveness."
   a. Truth	b. Lie	c. I don't know
5. "Only bats that feed on blood can transmit the rabies virus."
   a. Truth	b. Lie	c. I don't know
6. "Only the veterinarian can be sure whether or not an animal has rabies".
   a. Truth	b. Lie	c. I don't know
7. "All bats have the rabies virus and, therefore, we must kill them so we don't catch the disease".
   a. Truth	b. Lie	c. I don't know
8. "If I am bitten by an unknown animal, I should just rub alcohol on the wound and bandage the bite site."
   a. Truth	b. Lie	c. I don't know
9. "The main way to prevent rabies is to give rabies vaccine to domestic animals."
   a. Truth	b. Lie	c. I don't know
10. Which figure contains elements that are part of anger? Select an option.

a.Dog, bat, mosquito and tick	b. Dog, bat and horse	c. I don't know

11. What should we do when we find a bat at home? Select an option

a.Scare away or hit him with a broom	b. Isolate it with a bucket or box	c. I don't know

**AFTER CLASS**

**What did you think of the class? Answer the questions below**

1. Circle the face that defines how much you found this class **BORING or COOL.**

2. Circle the face that defines how much you found this class **DIFFICULT or EASY to learn**.

3. Circle the face that defines how **much you LEARNED** in this class.

4. Circle the face that defines how EXCITED you are **to talk about what you learned today to your family**.

5. Do you prefer to do activities alone or in a group?
   a. Alone
   b. In a group

**Now that you've played the games, answer the questions:**

Read the following statements, and in each of them, mark whether you consider it to be true or false. If you don't know the answer, check the "I don't know" option. It's okay not to know or make mistakes!

Read the following statements, and in each of them, mark whether you consider it to be true or false. If you don't know the answer, check the "I don't know" option. It's okay not to know or make mistakes!

1. Have you ever heard about anger?
   a. Yes	b. No	c. I don't know

**2.** "People, dogs and cats can get rabies".

a. Yes	b. No	c. I don't know

**3.** "There is no treatment or remedies as efficient for rabies, which is why almost 100% of people with the disease die."

a. Yes	b. No	c. I don't know

**4.** "The only symptom that people or animals with the disease 'rabies' have is aggressiveness".

a. Yes	b. No	c. I don't know

**5.** "Only bats that feed on blood can transmit the rabies virus."

a. Yes	b. No	c. I don't know

**6.** "Only the veterinarian can be sure whether or not an animal has rabies".

a. Yes	b. No	c. I don't know

**7.** "All bats have the rabies virus and, therefore, we must kill them so we don't catch the disease".

a. Yes	b. No	c. I don't know

**8.** "If I am bitten by an unknown animal, I should just rub alcohol on the wound and bandage the bite site."

a. Yes	b. No	c. I don't know

**9.** "The main way to prevent rabies is to give rabies vaccine to domestic animals".

a. Yes	b. No	c. I don't know

**10**. Which figure contains elements that are part of anger? Select an option.

a.Dog, bat, mosquito and tick	b. Dog, bat and horse	c. I don't know

**11**. What should we do when we find a bat at home? Select an option.

a.Scare away or hit him with a broom	b. Isolate it with a bucket or box	c. I don't know

.
