# Supplementary material for: School-based one health education shapes young minds and households to prevent zoonoses: A comparison of four teaching strategies
Source: One Health. 2026 Aug 13;23:101541. doi: 10.1016/j.onehlt.2026.101541 (PMC13519258; doi:10.1016/j.onehlt.2026.101541)
Supplement: Supplementary file 4 [file mmc6.docx]

### **Supplementary Material 6 – Focus group**

### Supplementary Material 6. Thematic synthesis of focus group findings with representative quotations from students in Grades 3 and 7.

| **Theme** | **Representative student statements** |
| --- | --- |
| Engagement with educational strategies | “I liked the one where we were playing with the dice and the little figures.” (Grade 3)  “The fun part of the board game is the competition. We learn a lot.” (Grade 7)  “I liked it less when using the board because I had to copy everything.” (Grade 7)  “I really liked the board game because it helps me memorize.” (Grade 7)  “The board is very boring.” (Grade 7)  “When we sit in groups… it becomes clearer because we see other points of view.” (Grade 7) |
| Knowledge acquisition and recall | “If you see a bat flying during the day, you should be careful.” (Grade 3)  “Not every bat has rabies.” (Grade 3)  “I told her she had to get vaccinated, go to the doctor…” (Grade 3)  “If you are bitten by a dog, you need to wash it with tap water and go to the doctor.” (Grade 7)  “Now I know which mosquito transmits leishmaniasis.” (Grade 7) |
| Knowledge sharing at home | “I was happy because I had never taught my parents anything before.” (Grade 3)  “I talked to my mother… and she bought a flea collar for the dog.” (Grade 7)  “I removed all the leaves from the ground so mosquitoes wouldn’t appear.” (Grade 3)  “I liked doing it with my mother… it felt like we were playing together.” (Grade 3)  “We teach at home… and reinforce what we learn with our parents.” (Grade 7) |
