# Supplementary material for: School-based one health education shapes young minds and households to prevent zoonoses: A comparison of four teaching strategies
Source: One Health. 2026 Aug 13;23:101541. doi: 10.1016/j.onehlt.2026.101541 (PMC13519258; doi:10.1016/j.onehlt.2026.101541)
Supplement: Supplementary file 5 [file mmc7.docx]

**Supplementary materials 1, 2, and 3**
**Figures**

Supplementary material 1. Application of the Latin square design (Sampaio, 2007) to organize the educational sessions and thematic content according to the teaching strategies evaluated and the topics covered.

Supplementary Material 2. Board of the “Sporotrichosis Game” used during the educational intervention. The game includes reward and penalty spaces designed to reinforce knowledge of sporotrichosis prevention and responsible pet ownership.

Supplementary Material 3. Examples of cards used in the “Sporotrichosis Game” during the educational intervention. The cards contain multiple-choice questions designed to assess and reinforce students’ knowledge of sporotrichosis transmission and related One Health concepts.
