# Supplementary material for: School-based one health education shapes young minds and households to prevent zoonoses: A comparison of four teaching strategies
Source: One Health. 2026 Aug 13;23:101541. doi: 10.1016/j.onehlt.2026.101541 (PMC13519258; doi:10.1016/j.onehlt.2026.101541)
Supplement: Supplementary file 6 [file mmc8.docx]

**Figure captions**

**Figures**

Figure 1. Teaching strategies used in the study, respectively A) board game, B) theater, C) group discussion, and D) board.

Figure 2. Mosaic plot of the standardized Pearson residuals* with the p-value of the association between "knowledge about zoonoses" and "assessment moment" during the school-based health education project

Figure 3. Relative frequency (%) with a 95% confience interval regarding correctly identifying the "One Health" concept by class during the project.

Figure 4. Boxplot of the number of correct answers before and immediately after the interventions, by teaching strategy used with children aged six to 10 and 11 to 14 years, respectively.
